# Remediation of Cadmium Stress in Strawberry Plants Using Humic Acid and Silicon Applications

**DOI:** 10.3390/life12121962

**Published:** 2022-11-23

**Authors:** Meral Dogan, Ibrahim Bolat, Sema Karakas, Murat Dikilitas, Gastón Gutiérrez-Gamboa, Ozkan Kaya

**Affiliations:** 1 Department of Horticulture, Graduate School of Natural and Applied Sciences, Harran University, 63050 Sanliurfa, Türkiye; 2 Department of Horticulture, Faculty of Agriculture, Harran University, 63050 Sanlıurfa, Türkiye; 3 Department of Soil Science and Plant Nutrition, Faculty of Agriculture, Harran University, 63050 Sanlıurfa, Türkiye; 4 Department of Plant Protection, Faculty of Agriculture, Harran University, 63050 Sanlıurfa, Türkiye; 5 Escuela de Agronomía, Facultad de Ciencias, Ingeniería y Tecnología, Universidad Mayor, Temuco 4780000, Chile; 6 Erzincan Horticultural Research Institute, Republic of Turkey Ministry of Agriculture and Forestry, 24060 Erzincan, Türkiye

**Keywords:** abiotic stress, biostimulation, proline, Rubygem, stomatal conductance

## Abstract

(1) Background: Strawberry plants are strongly affected by heavy-metal-contaminated soils, which affects plant growth, yield and fruit quality. The aim of this work was to study the effects of a combination and individual application of silicon (Si) and humic acid (HA) on growth and development of Rubygem strawberries exposed to cadmium (Cd) in greenhouse conditions. (2) Methods: Morphological, physiological and biochemical parameters, including minerals in roots and leaves were determined. (3) Results: Cd stress in strawberry plants decreased plant fresh and dry weight; leaf stomatal conductance; leaf relative water content and chlorophyll content; number of leaves; leaf area; leaf N, P and K levels; and root P, N, Mg, K and Ca contents. Cd increased membrane permeability, leaf temperature, proline levels and lipid peroxidation. Si and HA individual applications to strawberries mitigated the negative effect of Cd stress on biochemical, physiological, morphological and minerals parameters by decreasing membrane permeability, leaf temperature, proline levels and lipid peroxidation. (4) Conclusions: Our findings highlighted that applications of Si, HA and Si + HA were effective in conferring Cd tolerance in strawberry plants by upregulating their many morphological, physiological and biochemical properties and reducing Cd stress.

## 1. Introduction

Biotic and abiotic stresses are the major constraints in agriculture, and many metabolic and physiological changes occur in plant species under these conditions [1,2]. Heavy metals, such as chromium, cadmium, arsenic, lead, mercury, zinc, nickel and copper, are common pollutants in soils, are biologically toxic and could persist long-term in the soil environment [3,4,5]. The soil in various agricultural regions has been partially contaminated with heavy metals, resulting in the reduced availability of farmland [6,7,8]. Cadmium (Cd) is a relative rare mineral and is not found in nature in pure form; it occurs mainly in association with the sulfide ores of zinc, lead and copper [9]. Cd is not required for plant development, and it accumulates in the plant, causing significant harmful effects [10,11]. Irrigation pipes, flue gases produced by coal and industrial production, refined petroleum products and fertilizers used in seed and crop production, especially those containing phosphates are important sources of Cd, which negatively affects plant life [12,13,14]. Cd is quickly absorbed and transported by roots to other organs of the plant [15]. Cd accumulation in plants causes significant metabolic disorders in nitrogen and carbohydrate metabolism [13,15]. Cd inhibits photosynthesis, plant growth and seed germination, inactivates proteins and enzymes, closes stomata, increase water loss by transpiration, disrupts chlorophyll biosynthesis and affects plant nutrient distribution, absorption and plant–water relations [16,17].

Some agricultural methods have been developed to minimize the negative effects of heavy metal stress in agriculture. Humic acid (HA) and silicon (Si) are commonly used as mitigation techniques to improve plant yield in soils under heavy metal stress. HA affects the biological chemical availability and solubility of free heavy metal compounds in soil by converting them to organic form [18,19]. HA improve the availability of nutrients for plants, making root uptake easier, more regular and more consistent by regulating soil pH [20,21]. It has also been reported that HA positively affects cell membrane, stem cells, photosynthesis and phosphorus intake and contributes to optimum plant development due to its positive effects on yield and quality by ensuring the balance of mineral substances in the soil and plant [22]. Si regulates cell organelles and develops ionic bridges with heavy metals, reducing the harm to metabolism and increasing the durability and activity of cell organelles [23]. Si can be accumulated in the plant organs, improving plant development in plants under heavy metal conditions [23].

Strawberry is one of the most economically major berry fruits and its cultivation is important in terms of human health and nutrition due to its content of vitamin C and its strong antioxidant capacities [24]. Some authors have studied how to alleviate Cd stress in different plant species by applying HA and Si [25,26,27,28,29,30,31,32,33]; however, there have been no studies performed to assess the method of HA and Si combination to obtain enough tolerance to Cd stress effects. This work highlighted the effects of these applications on the attenuation of Cd stress effects on strawberry plants. Therefore, the goal of this present study was to determine the effects of HA and Si treatments applied individually or in a mixture to Rubygem strawberries growing under Cd stress on morphological, physiological, biochemical and leaf and root mineral parameters.

## 2. Materials and Methods

### 2.1. Plant Material and Treatments

**Figure 1:**
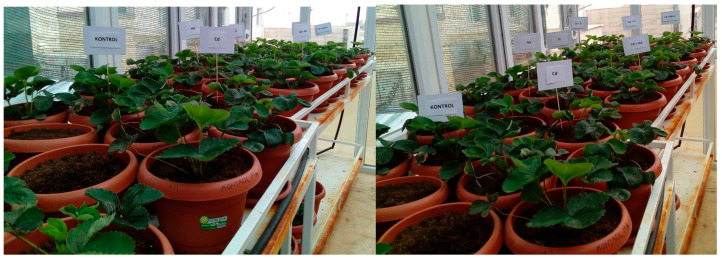
Strawberries plants subjected to different treatments in glass greenhouse conditions in the Department of Horticulture at Harran University.

The experiment was performed in a glass greenhouse belonging to the Harran University Faculty of Agriculture, Department of Horticulture (37° 10′ L.N; 38° 59′ L.E at 535 m a.s.l.) in 2017 season (Figure 1) using strawberry plants. The Rubygem (*Fragaria* × *ananassa* Duch.) strawberry variety is characterized by its short-day nature, produces high yields of moderate firm and is recommended for mild-winter climatic zones [34]. The temperature in the semi-controlled greenhouse varied between 34 °C during the day to 18 °C at night and relative humidity between 50 to 60%, with PAR (photosynthetically active radiation) between 860 to 1090 mmol m^−2^s^−1^. Rubygem fresh strawberry seedlings were grown in 5 L pots containing peat (Klasmann TS 1). Solution applications were begun on October 10, 2017, when the plants reached the stage of 4 to 5 emerging leaves. This trial was performed in a randomized block design, constituting four replications for each treatment and eight applications for a period of approximately 9 weeks. The root zone of the plants for all treatments every two weeks received 50% Hoagland’s solution manually. In group 1, pots did not receive any treatments and are the control. The control plants were only treated every two weeks with 50% Hoagland’s solution. In group 2, pots received humic acid (HA) at a concentration of 5 mM in Hoagland’s nutrient solution. In group 3, pots received silicon (Si) at a concentration of 5 mM in Hoagland’s nutrient solution. In group 4, pots received cadmium (Cd) at a concentration of 50 ppm in Hoagland’s nutrient solution. In group 5, pots received humic acid plus silicon (HA + Si) at a concentration of 5 mM + 5 mM in Hoagland’s nutrient solution. In group 6, pots received cadmium plus humic acid (HA + Cd) at a concentration of 5 mM + 50 ppm in Hoagland’s nutrient solution. In group 7, pots received cadmium plus humic acid (Si + Cd) at a concentration of 5 mM + 50 ppm in Hoagland’s nutrient solution. In group 8 pots received cadmium plus humic acid plus silicon treatment (Cd + HA + Si) at a concentration of 5 mM + 5 mM + 50 ppm in Hoagland’s nutrient solution. In this study, the concentration of 50 ppm Cd, 5 mM Si and 5 mM HA was chosen considering the results of previous researchers who were highly effective in studying the growth of strawberry seedlings [26,34,35,36]. The amount of water required for a plant in each plastic pot was determined according to the pots’ field capacity (FC). Since water is lost in pots by evapotranspiration, the pots were weighed at two-day intervals to determine the amount of water lost and thus, when to refill the consumed water. For the amount of water lost, the pot moisture mixture was filled to 100% of the field capacity. The amount of missing water was manually added to each pot. Subsequently, strawberry plants were harvested on 16 December 2017 for analysis. In addition, since the quality of light received by the plants is not always the same, the pots within each block were randomly moved from one place to another at partial intervals to eliminate the edge effect.

### 2.2. Plant Fresh and Dry Weight Measurements

After harvest, strawberry plants were carefully removed from the pots using distilled water. The total plant fresh weight (TPFW), total plant dry weight (TPDW), root fresh weight (RFW), shoot fresh weight (SFW), root dry weight (RDW) and shoot dry weight (SDW) per plant were measured following the protocol described in Bolat et al. [34].

### 2.3. Leaf Area, Total Number of Leaves and Relative Leaf Water Content Determinations

**Figure 2:**
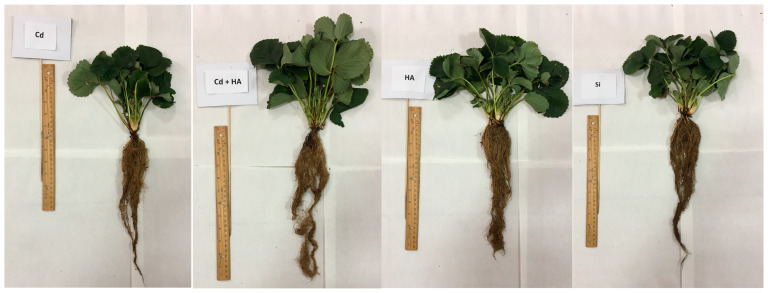
Leaves and roots of strawberries plants subjected to different treatments, such as cadmium (Cd), cadmium plus humic acid (Cd + HA), humic acid (HA) and silicon (Si) application.

The total number of plant leaves was calculated by counting each leaf collected from each strawberry pots. Mean leaf area per plant was measured based on the method documented by Klamkowski and Treder [37]. Briefly, trifoliate leaves were randomly selected and photographed (Figure 2). Plant leaf areas were determined through the ImageJ software program that allowed us to calculate the mean leaf area in cm^2^ using a length reference, as has been documented in different reports [37,38].

The leaf relative water content (LRWC) of the samples was determined after the plant leaves were kept in water for 24 h. In a ventilated oven, the samples were dried at 70 °C for 48 h. Sample value was performed as a percent, following the protocol described in Bolat et al. [34].

### 2.4. Stomatal Conductance, Chlorophyll Content and the Temperature and Membrane Permeability of Leaves

Stomatal conductance (SC) analysis in leaves was performed at the solar zenith (between 12.00–14.00 h), based on the protocol determined by some authors [39]. A leaf porometer (Decagon Devices Inc., Model SC-1, Steady-State Diffusion Porometer, Nebraska, WA, USA) was utilized to detect this parameter on plant leaves in the same positions between the middle and apical leaves of the samples.

Chlorophyll (Chl) values of plant leaves were detected by using a SPAD-502 Plus (Konica Minolta Optics, Inc., Tokyo, Japan). In leaves, the SPAD values were measured by two measurements detected on leaves in the same positions between the middle and apical leaves of the plant. The means regarding the SPAD reading values were registered and calculated by Bolat et al. [34].

Leaf temperature (LT) of plants was measured using an infrared thermometer on plant leaves in the same positions between the middle and apical leaves of the samples. LT values were determined on sunny days at noon when the clouds did not block the sun [34].

Membrane permeability (MP) was estimated using a relation of electrical conductivity and was expressed as a percentage considering the methodology documented by Lutts et al. [40]. Strawberry leaf samples were taken using leaf discs for each treatment. Leaf discs obtained from samples were placed in vials containing 10 mL of H_2_O and, for 30 min, boiled at 40 °C. Afterwards, the electrical conductivity of the samples solution (EC_1_) was measured. The final electrical conductivity (EC_2_) was detected using the leaf samples boiled at 100 °C for 10 min, and the relationship between EC_1_ and EC_2_ was utilized for MP calculation [40].

### 2.5. Proline and Malondialdehyde Determinations

Proline content was determined following the methodology published by some authors [34,41]. Thus, a 0.5 g leaf sample was ground in 10 mL of 3% sulfosalicylic acid. Afterwards, the samples were centrifuged for 10 min at 10,000 rpm. Subsequently, 2 mL of the supernatant was mixed within tubes with 2 mL of glacial acetic acid and 2 mL of the freshly prepared acid–ninhydrin. The tubes were incubated in a water bath at 90 °C for 1 h to allow a reaction. After this, tubes were extracted, utilizing 5 mL of toluene. Carefully, the toluene phase was collected. The sample absorbance was detected at 520 nm by a spectrophotometer (Shimadzu UV-1700, Kyoto, Japan). For the proline calculation, the standard curve was prepared using L-proline, and read values were expressed as µg g^−1^ of fresh weight.

For plant samples, lipid peroxidation (MDA) values were calculated based on the methodology expressed by some authors [34,42,43], using the thiobarbituric acid test. The sample absorbance value was read at 532 and 600 nm using a Shimadzu UV-1700 spectrophotometer.

### 2.6. Mineral Analysis of Leaves and Roots

Subsequent the strawberry harvest, leaves and roots were washed with distilled water. The samples were dried on blotting paper [34]. Then they were kept for 48 h in a oven set at 65–70 °C. The dried leaves obtained from the samples were ground in a mortar. The N content levels of samples was determined by the Kjeldahl procedure [34], whereas the Ca, Mg, and K values were determined with an atomic absorption spectrophotometer. The P content of samples was calculated using a spectrophotometer (Shimadzu UV-1700) [34]. Macro elements were expressed as percentages.

### 2.7. Data Analysis

Statistical analysis was performed with SPSS software (SPSS Version 23, IBM, 178 Chicago, IL, USA). The data were treated with an analysis of variance (ANOVA). The statistical difference of the mean values were detected utilizing Duncan’s test (*p* ≤ 0.05). Hierarchical clustering analysis (HCA) and principal component analysis (PCA) were conducted via the Software R (Version 4.1.1, R Foundation for Statistical Computing, Vienna, Austria)

## 3. Results

### 3.1. Strawberry Morphological, Physical and Physiological Analysis

Strawberry plant physical parameters, such as root fresh weight (RDW), shoot fresh weight (SFW), total plant dry weight (TPDW), total plant fresh weight (TPFW), shoot dry weight (SDW) and root fresh weight (RFW) were significantly affected by the treatments (Table 1). Strawberry plants subjected to cadmium (Cd) applications showed the lowest SFW, RFW, TPFW, SDW, RDW and TPDW contents. Contrary to this, strawberries subjected to individual applications of silicon (Si) and humic acid (HA) showed the highest levels of the physical determinations. Generally, HA and Si applications to strawberries plants exposed to Cd stress improved the levels of physical determinations, and the HA and Si individual treatments showed the best performance compared to HA plus Si applications.

**Figure 3:**
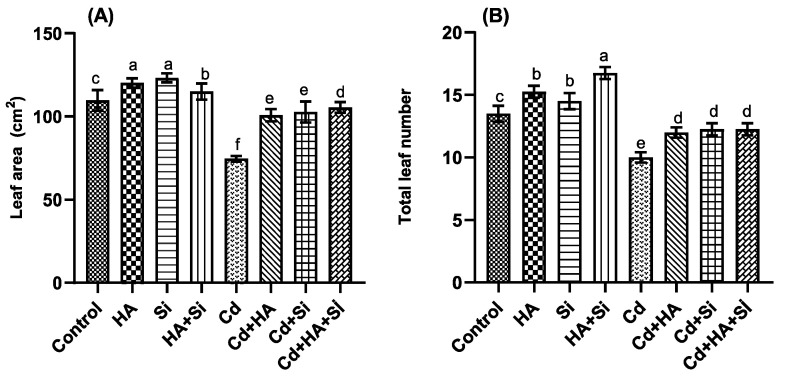
Effect of cadmium (Cd), silicon (Si) and humic acid (HA) applications in different ways on mean leaf area (**A**) and total leaf number (**B**) of strawberry plants compared to control. The bars indicate the means that are shown with their corresponding standard error. Different letters on each bar represent significant differences among applications (Duncan’s test, *p* < 0.05).

Cd stress markedly decreased the mean leaf area and total leaf number of plants, compared with the control and other treatments. On the other hand, the mean leaf area increased markedly with HA and Si applications and the total leaf area with HA + Si combinations (Figure 3).

**Figure 4:**
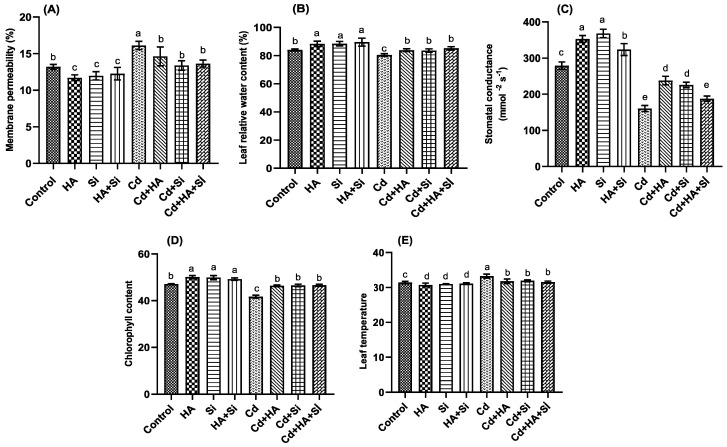
Effect of humic acid (HA), cadmium (Cd) and silicon (Si) applications in different ways on membrane permeability (**A**), leaf relative water content (**B**), stomatal conductance (**C**), chlorophyll content (**D**) and leaf temperature (**E**) of strawberry plants compared to the control. Leaf temperature was expressed in °C. The bars indicate the means, which are shown with their corresponding standard error. Different letters on each bar represent significant differences among applications (Duncan’s test, *p* < 0.05).

Strawberry plant physiological parameters, such as leaf temperature (LT), membrane permeability (MP), leaf relative water content (LRWC), stomatal conductance (SC) and chlorophyll content (Chl), were affected by the treatments (Figure 4). MP, LRWC, SC, Chl and LT levels were significantly affected by Cd application, whereas applications of Cd in combination with HA, Si and HA + Si mitigated their effects, except in the SC parameter. Strawberries plants subjected to HA, HA + Si and Si treatments presented the lowest MP and LT levels and the highest LRWC, SC and Chl contents.

### 3.2. Strawberry Biochemical Parameters

**Figure 5:**
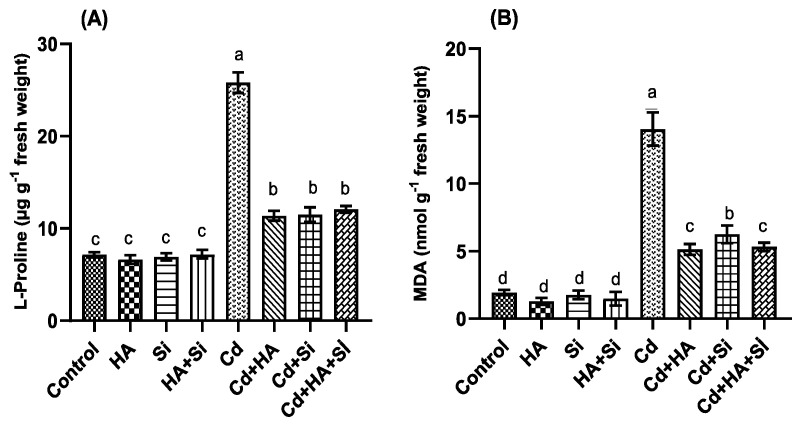
Effect of humic acid (HA), cadmium (Cd) and silicon (Si) applications in different ways on the proline (**A**) and manoldialdehyde-MDA (**B**) contents of strawberry plants compared to the control. The bars indicate the means that are shown with their corresponding standard error. Different letters on each bar represent significant differences (Duncan’s test, *p* < 0.05) among applications.

Strawberry plant biochemical parameters, such as proline and manoldialdehyde (MDA), were affected by the treatments applied (Figure 5). Proline and MDA levels were significantly affected by the Cd treatment, whereas Cd applications in combination with HA, Si and HA + Si considerably mitigated their effects. Strawberries plants subjected to HA, HA + Si and Si applications, as well as the control, presented the lowest MDA and proline contents.

### 3.3. Mineral Contents in Roots and Leaves

Strawberry root and leaf minerals, such as calcium (Ca), phosphorous (P), nitrogen (N), magnesium (Mg) and potassium (K) were significantly affected by the treatments (Table 2). Strawberry plants subjected to Cd applications showed the lowest N, P and K contents on leaves and Ca, P, N, Mg and K contents in roots. Contrary to this, strawberries subjected to individual treatments of silicon (Si) and humic acid (HA) showed the highest levels of most root and the leaf minerals, similar to control plants. In this way, HA and Si applications to strawberries plants subjected to Cd stress improved the levels of minerals in leaves and roots, except for Mg and Ca contents in leaves.

### 3.4. Hierarchical Clustering Analysis (HCA)

**Figure 6:**
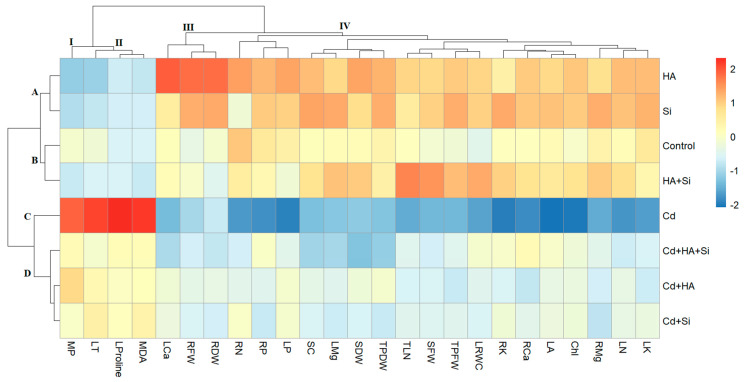
HCA for physiological, morphological, biochemical and mineral parameters in the Rubygem strawberry cultivar. Si: silicon, HA: humic acid, Cd: cadmium, HA + Si: humic acid plus silicon, Cd + HA: cadmium plus humic acid, Cd + Si: cadmium plus silicon, Cd + HA + Si: cadmium plus humic acid plus silicon, TPDW: total plant dry weight, RFW: root fresh weight, RDW: root fresh weight, TPFW: total plant fresh weight, LK: leaf potassium content, SDW: shoot dry weight, MP: membrane permeability, Chl: chlorophyll content, TLN: total leaf number, LA: leaf area, MDA: manoldialdehyde, SC: stomatal conductance, SFW: shoot fresh weight, LT: leaf temperature, LCa: leaf calcium content, LRWC: leaf relative water content, LP: leaf phosphorus content, LN: leaf nitrogen content, LMg: leaf magnesium content, RCa: root calcium content, RK: root potassium content, RN: root nitrogen content, RMg: root magnesium content, RP: root phosphorus content. A, B, C and D represent the groups of the treatments studied. I, II, III and IV represent the groups of parameters studied.

**Figure 7:**
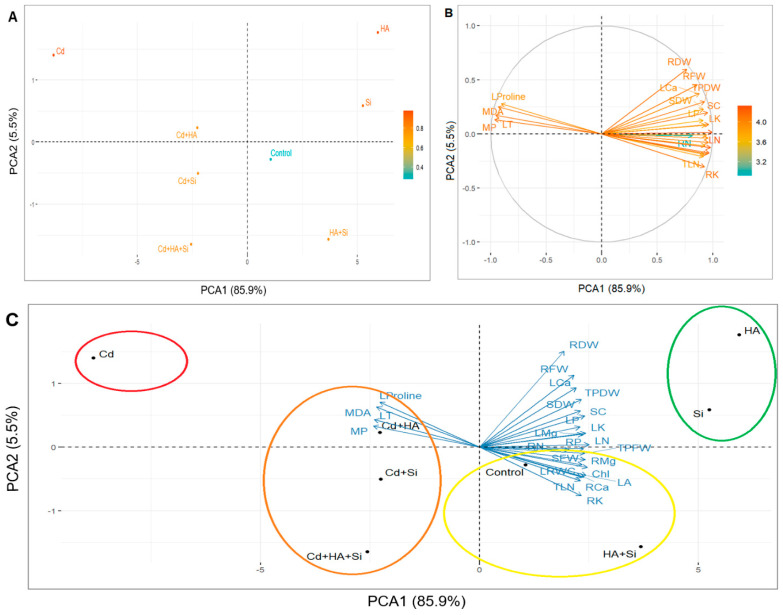
A loading plot of all the detected variables included in PCA for the biochemical, morphological, physiological, and mineral parameters in Rubygem strawberry cultivar. Treatments (**A**), biochemical, morphological, physiological, and mineral parameters (**B**), treatments and parameters (**C**). Si: silicon, HA: humic acid, Cd: cadmium, HA + Si: humic acid plus silicon, Cd + HA: cadmium plus humic acid, Cd + Si: cadmium plus silicon, Cd + HA + Si: cadmium plus humic acid plus silicon, RFW: root fresh weight, TPFW: total plant fresh weight, MP: membrane permeability, SFW: shoot fresh weight, RDW: root fresh weight, SDW: shoot dry weight, TPDW: total plant dry weight, LT: leaf temperature, LRWC: leaf relative water content, LA: leaf area, TLN: total leaf number, LP: leaf phosphorus content, MDA: manoldialdehyde, SC: stomatal conductance, LCa: leaf calcium content, LK: leaf potassium content, LN: leaf nitrogen content, LMg: leaf magnesium content, RK: root potassium content, Chl: chlorophyll content, RCa: root calcium content, RMg: root magnesium content, RN: root nitrogen content, RP: root phosphorus content.

HCA was performed, utilizing a total of 25 different parameters, including morphological, physiological and biochemical determinations (Figure 6). A heatmap graph of the HCA analysis shown that the treatments were divided into four different groups (Figure 7). Strawberries treated with HA and Si were in group A; strawberries treated with control and HA + Si were in group B; strawberries treated with Cd were in group C; and strawberries treated with Cd + HA, Cd + Si and Cd + HA + Si were in group D. Based on HCA physiological, morphological, biochemical and minerals parameters were grouped into four clusters. Leaf temperature, membrane permeability, proline and MDA were clustered in group I and II. These parameters showed the highest value in strawberries under Cd conditions or stress, and these values decreased with the effect of HA and Si. Leaf calcium, plant dry weight, root fresh weight and root dry weight were placed into group III. Leaf area; shoot fresh weight; total leaf number; shoot dry weight; plant fresh weight; stomatal conductance; leaf relative water content; chlorophyl content; and nitrogen, phosphorous, potassium, magnesium and calcium levels in roots and nitrogen, phosphorous, potassium and magnesium levels in leaves were clustered in group IV. The values of these parameters were low in strawberry plants subjected to Cd stress.

### 3.5. Principal Component Analysis (PCA)

PCA was performed using a total of 25 different parameters, including morphological, physiological and biochemical determinations (Figure 7). The co-observed variables in the PCA analysis were largely explained (85.9%). Cd treatment formed a negative group away from the control. In addition, Cd + HA, Cd + Si and Cd + HA + Si applications were closer to the control, but they also formed a negative group (Figure 6). A negative correlation was reported between Cd treatment and HA and Si treatments. On the other side, it was observed that the plants treated with HA + Si formed a group with the control, while the plants treated with HA and Si alone formed a different group contrary to Cd applications. This indicated that HA and Si individual applications had a positive effect on strawberry plants (Figure 7). Leaf area, root dry weight, leaf number, root fresh weight, shoot dry weight, shoot fresh weight, plant dry weight, plant fresh weight, stomatal conductance, leaf chlorophyll and leaf relative water content and minerals in leaves and roots are in the same group in plant-measured parameters towards HA and Si individual applications. In contrast, membrane permeability, proline, malondialdehyde and leaf temperature were in the opposite group regarding Cd stress (Figure 7).

## 4. Discussion

In this work, cadmium (Cd) stress in strawberry plants caused significantly negative effects on productivity, affecting the morphological, physiological, biochemical and mineral parameters of plants. Similar reports have been exposed by Muradoglu et al. [36], who showed that Cd applications to strawberry plants increased Cd levels on leaves and roots, resulting in a decrease in chlorophyll a and b in leaves and a considerable increase in malondialdehyde (MDA) contents. Trader et al. [44], showed that the increase of Cd content in soils resulted in a raise in Cd concentration in different strawberry tissues, especially in crowns and roots. In this report, the highest Cd accumulation was determined in strawberry leaf organs, which considerably affected strawberry productivity. Some studies on different plant species have reported that Cd-stressed plants were considerably affected in their vegetative parameters, which results in a considerable reduction in the dry and fresh weights of plants [36,45,46]. Rady [45] reported that Cd stress in bean plants produced a considerable decline in pigment parameters, vegetative growth, pod protein and green pod yield. Similar to our findings, this author also reported that Cd-stressed plants exhibited a considerable increase in MDA content and a decrease in relative water content and membrane stability index. In addition, Cai et al. [46] highlighted that Cd stress in rice resulted in a decrease in the levels of chlorophylls a and b, carotenoids and plant fresh weight. This report also reported that Cd stress increased the accumulation of Mn and Cu in shoots and roots and Zn content in shoots.

Several crops develop plant defense mechanisms to alleviate heavy metal stress [47]. Strawberries exposed to high levels of Cd disrupted their nutrient uptake (Table 2), as has been widely reported by some authors in different crops [44,46,48]. Przedpelska-Wasowicz et al. [49] reported that *Arabidopsis halleri* plants can translocate the excess of Cd to the aerial parts in high amounts. Strawberry plants under Cd stress considerably increased proline, lipid peroxidation, leaf temperature and membrane permeability and decreased leaf chlorophyll, stomatal conductance and leaf relative water content (Figure 3 and Figure 4). Muradoglu et al. [36] reported that strawberry plants subjected to Cd stress considerably increased lipid peroxidation and the activity of antioxidant enzymes, such as ascorbate peroxidase (APX), superoxide dismutase (SOD) and catalase (CAT). In this trial, the authors suggested that these determinations could be considered good indicators to determine Cd tolerance in strawberry plants. Some authors suggested that Cd stress in plants causes the production of reactive oxygen species (ROS), which in turn results in oxidative damage in plant tissues [47,50]. Some authors reported that ROS molecules caused oxidative damage in proteins, nucleic acids and lipids, which results in cell death due to membrane damage, lipid peroxidation and proline inhibition [47,50,51]. Studies conducted on pepper, eggplant and bean reported that Cd stress resulted in a considerable increase in membrane permeability, lipid peroxidation and proline [45,52,53]. Żabka et al. [54] reported that cellular responses to Cd toxicity consist of a series of interconnected biochemical reactions through DNA production and replication stress caused by the reactive oxygen species (ROS) damage. The biochemical reactions mentioned above allow the activatation of signal factors engaged in epigenetic adaptations, DNA repair systems and cell-cycle-control pathways [54].

Some studies have reported that humic acid and silicon improve plant growth, providing resistance against abiotic stress and reducing heavy metal stress [22,55,56,57]. Kim et al. [57] reported that silicon mitigated heavy metal stress by regulating endogenous phytohormones, silicon genes and P-type heavy metal ATPases. El-Okkiah et al. [56] stated that Cd stress in pea plants produced anatomical abnormalities, decreasing stomatal density. In addition, Cd stress induced oxidative damage in pea plants by increasing hydrogen peroxide (H_2_O_2_) and lipid peroxidation levels. Silicon application to these plants considerably reduced lipid peroxidation, protecting pea tissues against the oxidative stress caused by Cd toxicity [56]. Rostami et al. [55] noted that silicon applications altered Cd translocation and physiological trials of *Lallemantia royleana*, increasing vegatative growth, producing healthier plants under Cd stress. Silicon improves the water content of leaves due to the presence of a silicate layer that improves plant water balance by reducing water loss by transpiration [58]. Treder and Cieslinski [59] reported that silicon applications did not affect the Cd concentration in the roots; however, it significantly decreased the Cd concentration in the leaves and stems of strawberry plants. Humic acid promotes root growth in plants under osmotic stress by increasing water absorption and potassium uptake [22]. In addition, humic acid reduced the available content of heavy metals in soils by shifting bioavailable metals to stable phases that were less bioavailable [60]. Chen et al. [61] reported that humic acid mitigates Cd stress effects on photosynthetic properties and vegatative growth by increasing electron transport-related proteins, the expression of light-harvesting proteins, reaction center and rebuilt redox homeostasis and enhanced S metabolism in cells in lettuce. Similarly, it was reported that humic acid successfully mitigated Cd toxicity by modulating photosynthetic apparatus, antioxidant activity and the water status in wheat leaves. Therefore, humic acid could mitigate the negative effects of Cd stress on morphological, physiological and biochemical parameters in wheat plants [62]. The effects of HA, Si and NO treatments were also detected by some authors [34,63,64], who stated that their treatment promoted plant development and oxidative stress tolerance in plum, apricot and strawberry.

PCA and HCA analyses showed that there was a negative correlation between Cd treatment and HA, Si and Si + HA treatments. This shows that HA, Si and Si + HA applications are directly related to many parameters such as leaf area, root dry weight, leaf number, root fresh weight, shoot dry weight, shoot fresh weight, plant dry weight, plant fresh weight, stomatal conductance, leaf chlorophyll and leaf relative water content and minerals in leaves and roots under Cd stress in strawberry plants (Figure 6 and Figure 7). On the other hand, membrane permeability, proline, malondialdehyde and leaf temperature showed a negative relationship with Cd stress (Figure 6 and Figure 7). Previous works reported that Cd stress causes oxidative damage in plants [27,28,30,31,32,33], which is consistent with our findings. We noted that Cd stress in strawberry leaves increased membrane permeability, proline, malondialdehyde and leaf temperature content, implying that it causes oxidative damage in strawberry plants. It has also been explained by many studies that Si application improves plant tolerance by reducing Cd uptake and translocation [27,28,32,65]. In our findings, it has been highlighted that Si, HA and Si + HA applications play a desirable role in plant growth by causing beneficial effects on the mechanical strength of the plant under stress and thus, increase plant resistance.

## 5. Conclusions

Our study results indicated that applications of Si, HA and Si + HA upregulated many morphological, physiological and biochemical properties of strawberry plants and alleviated the negative effects of Cd on plant growth and development under Cd stress. Since this study was carried out only in pots under greenhouse conditions and not on Cd-contaminated soils, the present results may differ for strawberry plants grown in Cd-contaminated soils. Therefore, it is still necessary to further investigate different doses of Si, HA and Si + HA on the fruit yield and quality of strawberry plants grown in Cd-contaminated soils in the future research. However, the current results may provide some degree of theoretical basis for the application of Si, HA and Si + HA in open-field strawberry production.

## Figures and Tables

**Table 1:** Shoot, root and plant fresh and dry weights of strawberry plants (g plant^−1^).

	Shoot Fresh Weight	Root Fresh Weight	Total Plant Fresh Weight	Shoot Dry Weight	Root Dry Weight	Total Plant Dry Weight
Control	31.06 ± 0.31 b	5.90 ± 0.08 b	36.67 ± 0.38 b	11.04 ± 0.42 a	2.37 ± 0.14 b	13.93 ± 0.42 b
Humic acid (HA)	33.99 ± 0.95 a	9.42 ± 0.52 a	41.13 ± 1.18 a	11.79 ± 0.15 a	3.37 ± 0.21 a	15.30 ± 0.28 a
Silicon (Si)	34.20 ± 1.28 a	8.52 ± 0.27 a	42.12 ± 1.51 a	11.45 ± 0.17 a	3.11 ± 0.14 a	15.31± 0.30 a
Humic acid + silicon (HA + Si)	35.73 ± 0.99 a	6.43 ± 0.40 b	41.70 ± 0.68 a	11.56 ± 0.19 a	2.16 ± 0.04 b	14.11± 0.15 b
Cadmium	27.35 ± 0.44 c	4.86 ± 0.84 c	32.00 ± 0.40 c	10.17 ± 0.15 d	2.03 ± 0.06 b	11.50± 0.12 d
Cadmium + humic acid (Cd + HA)	29.74 ± 0.16 b	5.83 ± 0.24 b	34.54 ± 0.39 b	10.79 ± 0.27 b	2.22 ± 0.12 b	13.32± 0.26 b
Cadmium + silicon (Cd + Si)	29.94 ± 0.96 b	5.59 ± 0.94 b	35.51 ± 1.01 b	10.54 ± 0.20 c	2.07 ± 0.05 b	12.32 ± 0.18 c
Cadmium + humic acid + silicon (Cd + HA + Si)	29.58 ± 0.39 b	5.46 ± 0.18 b	35.49 ± 0.54 b	10.10 ± 0.24 d	2.01 ± 0.06 c	11.69 ± 0.20 e

Different letters in a column for a given factor represent statistically significant differences (Duncan test, *p* < 0.05).

**Table 2:** Mineral content of the leaves and root (% of dry weight) of strawberries plants subjected to humic acid (HA), cadmium (Cd) and silicon (Si) applications in different ways compared to control.

	Leaf Mineral Content					Root Mineral Content				
	N	P	K	Mg	Ca	N	P	K	Mg	Ca
Control	2.71 ± 0.05 a	1.34 ± 0.04 a	2.78 ± 0.03 a	0.40 ± 0.03 a	2.13 ± 0.04 c	2.07 ± 0.02 a	0.97 ± 0.02 a	2.27 ± 0.09 a	0.97 ± 0.02 a	0.79 ± 0.05 ab
HA	2.82 ± 0.03 a	1.42 ± 0.04 a	2.86 ± 0.02 a	0.43 ± 0.02 a	2.56 ± 0.02 a	2.10 ± 0.03 a	1.01 ± 0.01 a	2.33 ± 0.04 a	1.00 ± 0.05 a	0.88 ± 0.07 a
Si	2.81 ± 0.05 a	1.39 ± 0.03 ab	2.87 ± 0.04 a	0.45 ± 0.01 a	2.22 ± 0.01 b	1.97 ± 0.02 b	0.99 ± 0.02 ab	2.49 ± 0.14 a	1.03 ± 0.03 a	0.88 ± 0.05 a
HA + Si	2.78 ± 0.03 a	1.29 ± 0.03 b	2.73 ± 0.03 bc	0.44 ± 0.02 a	2.13 ± 0.02 c	2.03 ± 0.01 b	0.94 ± 0.03 b	2.42 ± 0.03 b	1.01 ± 0.01 b	0.85 ± 0.03 b
Cd	2.48 ± 0.08 c	1.14 ± 0.03 c	2.43 ± 0.04 e	0.34 ± 0.03 b	1.77 ± 0.02 d	1.85 ± 0.02 d	0.80 ± 0.01 d	1.91 ± 0.03 c	0.86 ± 0.03 d	0.61 ± 0.02 d
Cd + HA	2.65 ± 0.06 b	1.30 ± 0.02 b	2.58 ± 0.03 d	0.37 ± 0.01 b	2.05 ± 0.01 c	1.95 ± 0.02 c	0.89 ± 0.03 c	2.16 ± 0.08 b	0.91 ± 0.02 b	0.71 ± 0.04 c
Cd + Si	2.64 ± 0.05 ab	1.30 ± 0.03 b	2.64 ± 0.03 cd	0.36 ± 0.01 b	2.03 ± 0.02 c	1.99 ± 0.01 bc	0.87 ± 0.02 c	2.23 ± 0.07 b	0.90 ± 0.03 c	0.74 ± 0.05 a
Cd + Ha + Si	2.60 ± 0.04 b	1.27 ± 0.04 b	2.59 ± 0.04 d	0.35 ± 0.01 b	1.86 ± 0.02 d	1.94 ± 0.01 c	0.92 ± 0.01 c	2.25 ± 0.07 b	0.92 ± 0.01 b	0.80 ± 0.03 a

Abbreviations: humic acid (HA), silicon (Si), cadmium (Cd), magnesium (Mg) phosphorous (P), nitrogen (N), calcium (Ca) and potassium (K). Different letters in a column for a given factor represent significant differences (Duncan test, *p* < 0.05).

## Data Availability

Not applicable.
